# A Review on the Health Effects of Pesticides Based on Host Gut Microbiome and Metabolomics

**DOI:** 10.3389/fmolb.2021.632955

**Published:** 2021-02-08

**Authors:** Meng Zhou, Jiang Zhao

**Affiliations:** ^1^ College of Economics and Management, China Agricultural University, Beijing, China; ^2^ School of Mathematics and Statistics, Beijing Technology and Business University, Beijing, China

**Keywords:** pesticides, toxicity effects, gut microbiome, metabolomics, health effects

## Abstract

Due to their large number of applications, the pesticides pose potential toxicity risks to the non-target organisms. In recent years, the studies on the toxic effects of pesticides on non-target organisms, based on their gut microbiome and metabolome, have been continuously reported. As a dense and diverse microbial community, the gut microbiota in the mammalian gut plays a key role in the maintenance of host metabolic homeostasis. The imbalance in the gut microbiota of host is closely associated with the disturbance in the host's metabolic profile. A comprehensive analysis of the changes in the gut microbiota and metabolic profile of host will help in understanding the internal mechanism of pesticide-induced toxic effects. This study reviewed the composition and function of the gut microbiota of host, as well as the analysis methods and applications of metabolomics. Importantly, the latest research on the toxic effects of the exposure of pesticide to host was reviewed on the basis of changes in their gut microbiota and metabolic profile.

## Introduction

Pesticides are widely used as an important mean for agricultural production. However, the pesticide residues its excessive and unreasonable use, which seriously threatens the quality of agricultural products and the safety of ecological environment (Meng et al., 2020). At present, the pesticide residues have been widespread in many environmental media, such as food, water and soil (Yu et al., 2016; Silva et al., 2019). The pesticides can effectively enter non-target organisms, including humans, through a variety of ways, and bring potential health risks to them. A number of studies have shown that the long-term exposure to pesticides is often associated with diseases, such as cancer, hormonal disorders, asthma and allergies (Van Maele-Fabry et al., 2010). In addition, the long-term exposure to pesticides also has an impact on the offspring, causing reduction in their birth weight and increase in their mortality (Baldi et al., 2011; Meenakshi et al., 2012).

In recent years, the studies on host gut microbiome and metabolomics have attracted more attention (Zhang et al., 2015; Federici, 2019). The host intestinal microbiota is considered to be a virtual endocrine organ, which plays an important role in the maintenance of the physiological homeostasis of host (Ley et al., 2006; Flint et al., 2012). Generally, the gut microbiota of host is closely related to the host's metabolic profile. Metabolomics is based on the detection and analysis of small molecular metabolites, and mainly involves the changes in the body’s endogenous metabolites. Interestingly, many studies have shown that the exposure to multiple pesticides affects the composition of host's gut microbiota and metabolic profile (Zhao et al., 2016; Liu et al., 2017a; Wang et al., 2019a; Yan et al., 2020a; Meng et al., 2020). However, the role of the host intestinal microbiota and metabolic profile in the toxicity of pesticides induced in the non-target organisms is needed to be further.

In this study, the composition and function of gut microbiota, and the analysis methods and applications of metabolomics have been reviewed. Importantly, the effects of pesticides on the gut microbiota and metabolic profile of host were reviewed, which further emphasized on their key role in the pesticide-induced host toxicity.

## Gut Microbiome

### Host’s Gut Microbiota and Its Metabolites

A wide variety of microorganisms reside in the human gastrointestinal tract which are known as gut microbiota. Generally, the gut microbiota consists of about 500∼1,000 species, which mainly include anaerobic bacteria (Clemente et al., 2012). The most abundant phyla of the human intestinal microbiota are *Firmicutes* and *Bacteriodetes*. In addition, *Proteobacteria*, *Verrumicrobia*, *Actinomycetes*, *Fusobacteria* and *Cyanobacteria* are also relative abundant in the human intestines (Qin et al., 2010; Huttenhower et al., 2012). In adults, there are about 10^14^ bacterial cells in their intestines. They have a huge genome and participate in many physiological and biochemical activities of the host (Methe et al., 2012). The gut microbiota is necessary for the development and differentiation of host intestinal epithelium and immune system, and provides protection against the opportunistic pathogens. In addition, the gut microbiota plays a key role in the maintenance of tissue homeostasis (Smith et al., 2007).

**FIGURE 1:**
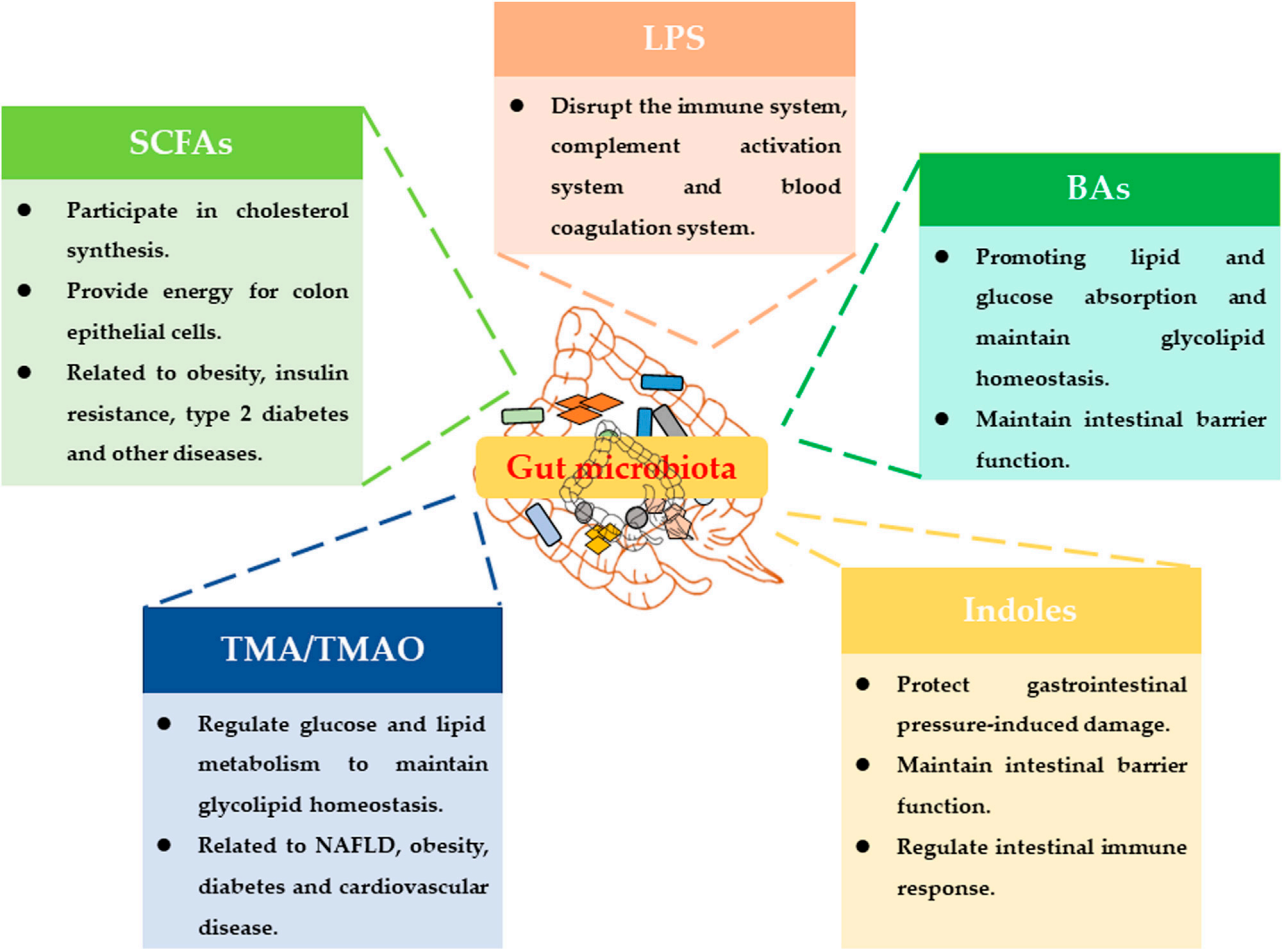
Main metabolites of gut microbiota and their physiological functions.

On the other hand, the metabolic capacity of the gut microbiota greatly exceeds the metabolic capacity of human cells. More and more studies have shown that the regulation of host physiological functions by gut microbiota was basically related to the metabolites of gut microbiota (Nicholson et al., 2012). Current research has reported many metabolites of gut microbiota, such as lipopolysaccharides (LPS), trimethylamine (TMA), short-chain fatty acids (SCFAs), bile acids (BAs) and indoles (Figure 1) (Tang et al., 2017).

LPS is a unique component of the cell wall of gram-negative bacteria. LPS will be released into the intestinal environment after the bacteria die and lyse, and enter the blood circulation system. It can disrupt the host's immune system, complement activation system and blood coagulation system (Carpino et al., 2020). The choline, phosphatidylcholine and L-carnitine in food can be metabolized by the intestinal flora to produce TMA. TMA can be converted into trimethylamine oxide (TMAO) under the oxidation of Flavin containing monooxygenase 3 (Fmo3). TMAO is an important risk factor for cardiovascular and cerebrovascular diseases such as atherosclerosis and thrombosis (Roberts et al., 2018). BAs play an important role in the body’s energy metabolism, and their main function is to promote energy digestion and absorption (Wahlstrom et al., 2016). SCFAs are foods that cannot be metabolized by the intestinal bacteria fermented by the host and produce organic fatty acids with a carbon chain length of less than 6. It can regulate host energy metabolism by activating GPR41 and GPR43 (Lavelle and Sokol, 2020). Tryptophan can produce many indoles through the metabolism of gut microbiota. Indoles may maintain the homeostasis of the intestinal barrier and regulate the intestinal immune response through AHR (Lavelle and Sokol, 2020).

### The Function of the Host Gut Microbiota

#### Barrier and Immunity

The intestine is not only a place to absorb nutrients, but also an important defense barrier for the body against the invasion of pathogenic bacteria. The intestinal barrier maintains the stability of intestinal immunity while defending against the exogenous infections. The gut microbiota plays an important role in the maintenance of the function of intestinal barrier. It promotes the development and maturation of intestinal epithelial cells. The intestinal epithelial cells play an important role in keeping the host safe from being invaded by foreign bacteria. The products produced by the metabolism of gut microbiota in the intestine can provide nutrients for the growth and development of intestinal epithelial cells, thereby maintaining their integrity. Previous studies have shown that the number of intestinal epithelial cells in a sterile mice was significantly lower than that in the normal mice. Therefore, the sterile animals are more susceptible to infections (Baba et al., 1991; Taguchi et al., 2002). In addition, the gut microbiota in healthy humans can also inhibit the invasion of foreign pathogenic bacteria, thereby protecting the host. Under normal circumstances, the composition of gut microbiota in a host is relatively stable. However, once the host is subjected to a strong external interference, the balance of gut microbiota in the host’s body is disturbed, resulting in an increase in the number of pathogenic bacteria and endangering the body’s health (Heyman et al., 1989; Sekirov et al., 2008; Ferreira et al., 2011). On the other hand, the gut microbiota can also provide signal to the host's immune system. The normal gut microbiota can induce the activation of β and T cells by affecting the expression of a series of different immune pathway-related genes, thereby assisting the host to resist against the foreign invading pathogens (Shanahan, 2000). Previous studies have shown that the lymph nodes in the mesentery of sterile animals are fewer and smaller than the normal animals, and the level of antibodies secreted by them is also significantly lower than that of the normal animals (Alam et al., 1994; Round and Mazmanian, 2009). In a normal host, the specific gut microbiota can inhibit inflammation, while some bacteria in the gut microbiota can become pathogenic under certain conditions and cause inflammation (Podolsky, 2002). The study of Ivanov et al. found that the *Bacteroidetes* could regulate the number of TH17 cells in the small intestine (Podolsky, 2002). This indicated that the specific bacteria in gut microbiota regulated the inflammatory response in intestine. In addition, the previous studies have also shown that antibiotics could reduce the symptoms of intestinal inflammation in mice (Videla et al., 1994). In short, the gut microbiota plays an important role in the regulation of barrier and immune functions in the host.

#### Nutrition and Metabolism

The main function of human digestive system is to digest and absorb food. From the traditional point of view, the colon is mainly considered as an organ for the absorption of water and minerals. However, people gradually discovered that the gut microbiota in colon further digests the unabsorbed carbohydrates in the upper digestive tract. The gut microbiota has completely different biochemical pathways and various enzyme systems than the host, which enables it to carry out the complex metabolic activities. On the one hand, the metabolic activities of gut microbiota provide energy essential for the growth and reproduction of microbiota. It can provide more energy for the host as well. Previous studies have shown that, in the same calorie-fed normal and sterile mice, the weight of normal mice was significantly higher than that of sterile mice (Backhed et al., 2004). In a recent study, the composition of gut microbiota of obese mice was found to be completely different from the composition of gut microbiota of thin mice (Ley et al., 2006). Subsequent studies found that the gut microbiota in obese mice increased the energy absorption capacity of host (Turnbaugh et al., 2006). In the cecum and colon, due less intestinal juice and slow bowel movement, the microorganisms grow rapidly and carry out fermentation to produce a large amounts of short-chain fatty acids (SCFAs) (Macfarlane et al., 1992). The SCFAs, especially acetate, propionate and butyrate obtained by the fermentation of gut microbiota, are of great importance for the physiological functions of host. About 95% of the SCFAs are absorbed and used by the host (Koh et al., 2016). Among them, the acetate can enter the peripheral circulation and gets metabolized by host to provide more energy for the host (Wong et al., 2006; Koh et al., 2016). The acetate can also affect the metabolism of fats and carbohydrates in liver. It also affects the metabolism of cholesterol and lipids in liver by activating the cytoplasmic acetyl-CoA synthase. In addition, most of the propionate is absorbed and utilized by the host's liver (Wong et al., 2006), which inhibits the promotion of acetate in fat synthesis, thereby regulating the host's fat synthesis (Wolever et al., 1995).

## Metabolomics

### Metabolomics and Its Analysis Methods

**FIGURE 2:**
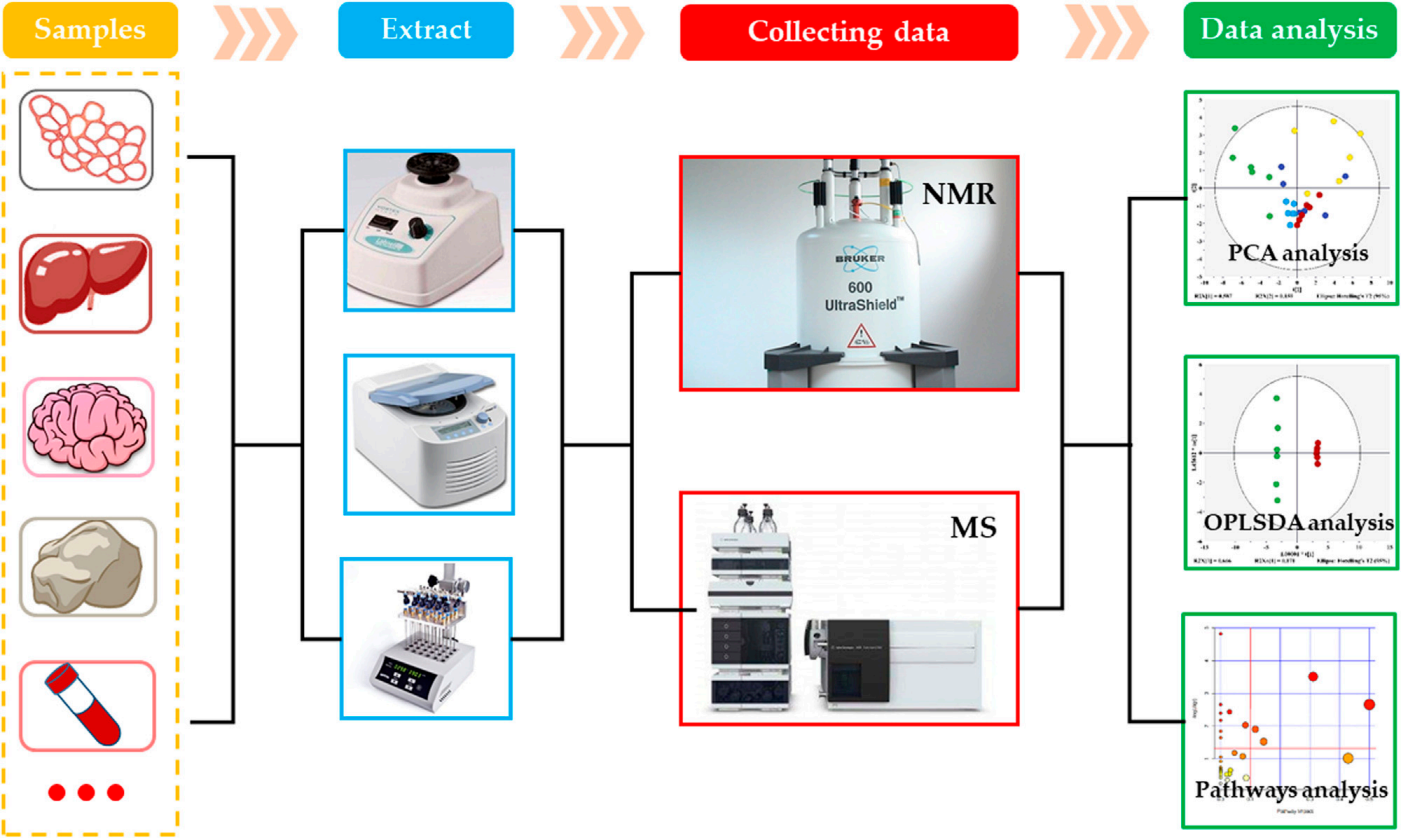
Routine procedure of metabolomics.

As an important branch of systems biology, the metabolomics is an emerging omics technology, following the proteomics, transcriptomics and genomics (Dettmer et al., 2010). The concept of metabolomics was first proposed by Nicholson et al. in 1999. In the following 20 years, the metabolomics developed rapidly and became one of the most active fields in life science research. The metabolomics is the study of physiological and metabolic changes in organisms using the qualitative and quantitative analysis of metabolites having relatively small molecular weights produced during the metabolism in organisms. Generally, the metabolomics can be divided into targeted and non-targeted metabolomics. The targeted metabolic analysis is a biased metabolomics analysis technology, which mainly focuses on the quantitative analysis and determination of the target metabolites of interest, and aims to study one or more metabolic pathways. However, the non-targeted metabolic analysis is an unbiased metabolomics technology that comprehensively analyzes all small molecular metabolites and studies the dynamic changes in various metabolites (Roberts et al., 2012). At present, there are various analytical techniques used for the metabolomics analysis, of which the most commonly used are nuclear magnetic resonance (NMR) and mass spectrometry (MS) (Figure 2) (Dunn et al., 2011).

NMR technology is one of the earliest analytical methods used in metabolomics. It uses the transition in energy level of atomic nuclei in electric field to analyze the chemical composition and spatial structure of substances, and makes the high-throughput analysis of small molecular metabolites (Li et al., 2015). At present, the most commonly used forms of NMR are hydrogen nuclear magnetic resonance spectroscopy (^1^H-NMR), carbon nuclear magnetic resonance spectroscopy (^13^C-NMR), nitrogen nuclear magnetic resonance spectroscopy (^15^N-NMR), and phosphorus nuclear magnetic resonance spectroscopy (^31^P-NMR), of which the ^1^H-NMR and ^13^C NMR are the most widely used NMR spectroscopies in metabolomics. The NMR can detect most of the endogenous metabolites in organisms, and can obtain a wealth of information about the tested samples. It provides stable results with good repeatability. In addition, the NMR has relatively simple requirements for the pretreatment of samples, and can perform unbiased and non-destructive analysis and detection of samples (Li et al., 2015). However, the NMR also has some shortcomings, which are mainly manifested in its insufficient sensitivity and resolution that cause difficulty in the detection of metabolites having low concentrations. Besides, the NMR requires a large sample volume, and gives overlapping metabolite peaks that are difficult to identify.

MS is one of the most widely used analysis methods in metabolomics. Currently, the liquid chromatography-mass spectrometry (LC-MS) and gas chromatography-mass spectrometry (GC-MS) are commonly used (Dunn and Ellis, 2005; Dettmer et al., 2010). These types of analysis method uses chromatography for the separation of metabolites and mass spectrometry for their identification, which overcome the limitations of NMR sensitivity and resolution. Among them, the LC-MS can analyze compounds having large polarity and relative molecular mass and thermally unstable or non-volatile compounds. Its sample pretreatment is relatively simple and without derivatization. Its disadvantage is that it takes long analysis time and lacks a corresponding database. On the other hand, the main advantage of GC-MS is that it has a standard database, which makes the quality of metabolite more convenient. It has higher sensitivity, especially for the volatile small molecular metabolites. However, this method still has the limitation of a small detection range. Although, a series of derivatization treatments can be carried out for the thermally unstable, non-volatile or polar metabolites to expand the detection range, the sample pretreatment is more complicated. In short, for the small sample amount and exploring the influence of chemicals on certain specific pathways, it is more appropriate to use the MS for analysis.

Due to the limitations in existing analysis platforms, they are not suitable for the analysis of all metabolites in a biological sample. By combining various single analysis techniques to form a joint technique, its application range becomes wider, and provides accurate information about a specific metabolome. Therefore, in the field of metabolomics research, several analytical techniques are often used to study the metabolites. There is a continuous developmental trend in the modern analytical techniques, which will promote the metabolomics research. At present, the most common example of the combination of multiple analysis techniques is the combination of NMR and MS. This method can make the full use of the advantages of various techniques for the identification of metabolites in organisms to the greatest extent. This combined method is not only used in the pesticide environmental toxicology research, but also provides an opportunity for the integration of multiple omics in system biology (Crockford et al., 2006).

After obtaining the original data by measuring the samples through the above-mentioned analysis techniques, the data needs to be preprocessed to make it suitable for the multivariate analysis. Depending on the technique used, the processing steps are slightly different (Wang et al., 2018b). The data preprocessing steps mainly include noise filtering, overlapping peak resolution, peak picking, peak alignment, baseline correction and metabolite identification, etc. (Lukowicz et al., 2018). The multivariate analysis methods in metabolomics are divided into unsupervized and supervised learning methods. The unsupervized learning methods only classify the samples on the basis of differences in the original data, but do not provide the information about sample classification. In the supervised learning methods, a mathematical model is established on the basis of original data to maximize the distinction between groups while providing the information of classification of the samples. The commonly used unsupervized learning method in metabolomics includes principal components analysis (PCA), while that of supervised methods include partial least squares-discriminant analysis (PLS-DA) and orthogonal bias least squares-discriminant analysis (OPLS-DA) (Wang et al., 2018a). According to the pattern-recognition results, the variables, which contribute to the classification, are screened out as metabolites with significant changes, and then some network tools and public databases, such as MetaboAnalyst (https://www.metaboanalyst.ca/), HMDB (the human metabolome database) and KEGG (Kyoto encyclopedia of genes and genomes), etc. are used to interpret the biological information of metabolites. Subsequently, the *p* value and pathway influence value (PIV) are used to find the significantly affected metabolic pathways, and the mechanism of toxicity can be explored in-depth through the changes in metabolic pathways.

### Applications of Metabolomics

#### Application of Metabolomics in Disease Diagnosis and Drug Development

Metabolomics has been playing an increasingly important role in the basic research and clinical applications, and has been widely for studying the different functional states and diseases of various organisms. Li et al. collected the blood samples of 150 patients with severe obstructive coronary heart disease (CHD) and 150 healthy controls for the metabolomics analysis using ultra-high performance liquid chromatography-quadruple time-of-flight mass spectrometry (UHPLC-QTOF/MS). The study found significant changes in the metabolites of 105 CHD patients. In addition, six metabolites, including palmitic acid, linoleic acid, 4-pyridoxic acid, phosphatidylglycerol, carnitine and lithocholic acid, had a strong correlation with the CHD (Li et al., 2017). Zhao et al. analyzed the metabolites in the urine samples of 75 patients of essential hypertension and healthy controls using UHPLC-QTOF/MS technology. According to the PLS-DA pattern-recognition analysis, the significant changes in the metabolic pathways involving the metabolism of amino acids, fatty acids, steroid hormones, biosynthesis, etc. were screened out (Zhao et al., 2018). In addition, a metabolomics study of the lung adenocarcinoma cells found that the uridine monophosphate (UMP), uridine diphosphate (UDP), adenosine diphosphate (ADP), malic acid, malonyl-CoA, 22 metabolites, including nicotinamide adenine dinucleotide (NAD) and coenzyme A were abnormally expressed, which involved the multiple metabolic pathways, such as nucleotide metabolism, urea cycle, tricarboxylic acid cycle and glycerophospholipid metabolism (Wang et al., 2019b). In addition, the metabolomics also showed a unique role in clarifying the function of drugs and revealing their mechanisms (Everett et al., 2013; Pinu et al., 2019). A metabolomics study was carried out after culturing the liver cancer cells with new potential anticancer drug, Natrin, which revealed that the metabolism of liver cancer cells was affected by the action of Natrin, and identified 13 metabolites as potential biomarkers associated with the Natrin-induced apoptosis (Lu et al., 2019). A large number of studies have shown a wide range of application of metabolomics technology in medical research, and has revealed the mechanism of different diseases and drug actions on a large scale.

#### Application of Metabolomics in Food Science Research

In the food science studies, metabolomics is used to study the mechanism of action of the active ingredients in food and the effect of food nutrients on metabolism in the body. The metabolomics technology has enriched the study methods of food science and has organically combined the food science with medicine. A recent study found that the extract of blueberry could activate the BA receptors TGR5 and FXR by affecting the metabolism of host’s bile acids. The activation of TGR5 and FXR improves the lipid metabolism in adipose tissue and liver, thereby reducing obesity and improving metabolic syndrome (Guo et al., 2019). Another study found that the citrus extract (PMFE) rich in polymethoxy flavonoids could effectively improve the MetS-induced by high-fat diet (HFD) and reduced the enteral malnutrition by regulating the BCAA levels in the host's serum and feces (Zeng et al., 2020)**.** In addition, the metabolomics technology is also widely used in the identification of food quality. As early as 2002, Brescia et al. analyzed the metabolic profile of 41 red wines from southern, central and northern Apulia, Italy, using NMR. With the help of the PCA, the wines of these three regions could be clearly distinguished. The main substances that caused differences in the wine regions were amino acids (Brescia et al., 2002). Cho and others used the NMR technology and PCA method to analyze the metabolic profiles of matsutake at four levels in Korea. The research results showed that the difference in the content of choline, trehalose, threonine, leucine/isoleucine, succinic acid, alanine and fumaric acid was the main reason for the differences in the grades of matsutake (Cho et al., 2007).

#### Application of Metabolomics in Environmental Toxicology

The metabolomics has been widely used in environmental toxicology studies to determine the changes in the physiological and biochemical indicators of host and predict the mode of action of the chemical substances (Lankadurai et al., 2013; Yuan et al., 2019). The metabolomics can provide an effective method for the further clarification of the toxic effects of environmental compounds. Wang et al. investigated the effects of triphenyl phosphate (TPP) and its main metabolite diphenyl phosphate (DPP) on the metabolic profile after the neonatal exposure from postnatal days using ^1^H-NMR-based metabolomics. The study found that there were dose- and sex-specific responses of adult mice after exposure to TPP and DPP(Wang et al., 2018a). In addition, the previous studies have found that the perinatal exposure to BPA, BPS, BPF, and BPAF could cause age-related metabolic effects in female mice offspring using ^1^H-NMR-based serum metabolomics analysis (Meng et al., 2019b). Jordan et al. used the NMR metabolomics method to study the toxic effects of environmental endocrine disruptors, such as nonylphenol, dioctyl phthalate, and bisphenol propane on goldfish. It was found that the nonylphenol mainly interfered with the metabolism of body's glucose, lipid and nucleic acids, dioctyl phthalate affected the metabolism of glucose and lipids, and bisphenol propane affected the glucose metabolism (Jordan et al., 2012). Kalkhof et al. studied the toxic effects of B(a)P on mouse liver cancer cells and found that the expression of 163 metabolites changed significantly after B(a)P infected the mice liver cancer cells. These metabolites were mainly involved in the metabolism of lipids, amino acids and glucose in the cells (Kalkhof et al., 2015).

#### Application of Metabolomics in the Research of Gut Microbiota

Metabolomics is widely used in the study of gut microbiota to determine changes in the metabolites of gut microbiota. By accurately detecting and analyzing the metabolites of gut microbiota, looking for differences and changes in the metabolome of gut microbiota, the metabolic state of the host can be more directly presented, which is of great significance for studying the physiological functions of gut microbiota (Natividad et al., 2018; Zierer et al., 2018; Huang et al., 2019). Zheng et al. found a new gut microbiota metabolite N, N, N-trimethyl-5-aminovaleric acid (TMAVA) by non-targeted metabolomics, which could aggravate liver steatosis by inhibiting butyl betaine hydroxylase (BBOX) (Zhao et al., 2020). Liu et al. conducted a genome-wide association study and serum metabolomics analysis on a group of obese young Chinese. The study determined that obesity-related gut microbial species are related to changes in circulating metabolites (Liu et al., 2017b). In addition, a targeted metabolomics study based onBAs found that blueberry extract can improve diet and genetically induced metabolic syndrome by regulating the BAs pathway (Guo et al., 2019).

## Study of the Toxicity Effects of Pesticides Based on the Gut Microbiome and Metabolomics

The increasing use of pesticides year by year has attracted more attention toward their toxic effects on non-target organisms in the environment. Interestingly, many recent studies have shown that the gut microbiota plays a key role in the toxic effects of pesticides (Yuan et al., 2019; Meng et al., 2020). In addition, many studies have proved that the gut microbiota is essential for the maintenance of host metabolic homeostasis, such as energy, amino acid and bile acids (Dai et al., 2011; Ma et al., 2018). Importantly, the gut microbiota and metabolic disorders of host are associated with a variety of diseases, including obesity, diabetes and colon cancer (Laurans et al., 2018; O’Mahony et al., 2015). Similarly, studies have focused on the symbiosis of gut microbiota and metabolic disorders with the exposure to pesticide in order to further explore the toxicity mechanism of pesticides on non-target organisms. Organochlorine pesticides (OCPs), which were banned in 1970s and 80s, are still widely detected in environment in recent years (Maisano et al., 2016). The chronic exposure of p, p’-DDE and β-HCH could significantly alter the relative abundance and composition of gut microbiota in mice, increasing the relative abundances of *Firmicutes* and *Proteobacteria* and decreasing that of *Bacteroidetes*, *Verrucomicrobia*, *Actinobacteria, Candidatus* and *Saccharibacteria*. In addition, the metabolism of bile acids in mice was affected with the exposure of p, p’-DDE and β-HCH. Specifically, the p, p’-DDE and β-HCH increased the contents of cholic acid (CA), taurocholic acid (TCA), glyco-cholic acid (GCA) deoxy-cholic acid (DCA), taurodeoxycholic acid (TDCA), glycodeoxycholicacid (GDCA), lithocholic acid (LCA), taurolithocholic acid (TLCA), and glycolithocholic acid (GLCA) and decreased that of the β-MCA (Liu et al., 2017a). In addition, a study on endosulfan sulfate showed that the treatment with endosulfan sulfate alleviated the HFD-induced obesity in mice by affecting the gut microbiota, lipid metabolism and glucose homeostasis (Yan et al., 2020a). Studies on the toxicity of chlorpyrifos, an organ thiophosphate pesticide has been widely reported. Zhao et al. used the high-throughput sequencing and NMR based metabolomics approaches to study the toxic effects of chlorpyrifos on the gut microbiome and urine metabolome in mice. The results indicated that the exposure to chlorpyrifos led to the intestinal inflammation and abnormal intestinal permeability by altering the composition of gut microbiota and disturbing the urine metabolism profiles (Zhao et al., 2016). In addition, the exposure to chlorpyrifos also led to the imbalance in gut microbiota and liver metabolism, which ultimately led to an increase in the mucous volume in intestine and oxidative stress in zebrafish (Wang et al., 2019a). The perinatal exposure to nitenpyram caused an imbalance in the gut microbiota and fecal metabolites in offspring female mice, causing an increase in the abundance of *Akkermansia*, and a decrease in the abundance of *Desulfovibrionaceae* and *Lactobacillus*. In addition, the metabolites, mainly related to the metabolism of purine and BCAAs and TCA cycle in feces were changed, which ultimately led to an increase in the consumption of host energy (Yan et al., 2020b). In addition, the toxic effects of imazalil have also been reported. The exposure to imazalil not only caused an imbalance in the gut microbiota, but also interfered with the liver metabolism in zebrafish. The analysis of gut microbiome showed an increase in the abundances of Fusobacteria and Firmicutes and increase in that of Bacteroidetes after being exposed to imazalil. In addition, the hepatic metabolomics was analyzed using GC/MS-based technology. The results found that the pathways of TCA cycle and metabolism of glucose, amino acids, lipids and nucleotide showed a significant change after imazalil exposure (Jin et al., 2017). Similarly, the short-term pro-paraben exposure also caused disturbances in the gut microbiota and liver metabolic profile of adult male zebrafish (Zhang et al., 2019). Wu et al. explored the effects of exposure to propamocarb on the gut microbiota and metabolism in mice. The 16S rRNA gene sequencing revealed alteration in the overall microbial structure after exposure to propamocarb. In addition, the ^1^H-NMR analysis showed that a total of 20 fecal metabolites, mainly including succinate, SCFAs, bile acids and trimethylamine, were affected (Wu et al., 2018). Moreover, the previous studies showed the impact of the exposure of penconazole and its enantiomers on gut microbiota and metabolic profile in mice. The results of the study found that the chiral pesticide penconazole could cause the selective disturbance of gut microbiota, and serum metabolic profile (Meng et al., 2019a). Current studies have confirmed that the exposure to pesticides can cause the dysbiosis of gut microbiota and metabolic disorders in the host. However, the correlation between the gut microbiota and metabolic profile is needed to be further investigated. The role of the changes in the gut microbiota and metabolic profile of host in the toxicity of pesticides is still not clear. The gut microbiota might change specific metabolites or specific metabolic pathways, further adversely affecting the host. Therefore, their roles in the toxic effects induced by pesticides are needed to be studied in-depth.

## Perspectives

In recent years, the gut microbiota of host has received more attention. Generally, the dysbiosis of gut microbiota often occurs simultaneously with the metabolic disorder of host. The studies on the health effects of host based on the changes in gut microbiota and metabolic profile have been continuously reported. In the past few decades, the pesticides have been widely used, and their toxic effects on the non-target organisms have received increasing attention. In particular, some studies have confirmed the destructive role of pesticides on the gut microbiota and metabolic profile of host. Unfortunately, most of these studies have only established a simple correlation between the pesticides and host gut microbiota and metabolic profile, while the key roles of the gut microbiota and metabolic profile host in the pesticide-induced toxic effects are still not focused. In particular, some studies have shown that some specific bacteria in gut microbiota can regulate specific metabolites or specific metabolic pathways, further affecting the host health. However, the current studies on the toxic effects of pesticides have not established a clear relationship between the specific pesticides and specific gut microbiota or metabolites. On the other hand, the key role of the gut microbiota and metabolic profile of host in the pesticide-induced toxic effect is needed to be further confirmed either by the transplantation of microbiota or the dietary supplementation of specific metabolites. In summary, this study reviewed the studies on the toxic effects of pesticides based on the gut microbiome and metabolomics of host. This review further emphasized on the role of changes in the gut microbiome and metabolomics of host in the pesticide-induced host toxic effects.
